# From first cigarette to nicotine dependence: Conventional Smoking among adolescents in Tunisia

**DOI:** 10.1192/j.eurpsy.2026.10857

**Published:** 2026-07-31

**Authors:** Z. Mallek, M. Trigui, Y. Mejdoub, E. Mziou, M. Baklouti, F. Ben Dhaou, J. Jdidi, S. Yaich, M. Kassis

**Affiliations:** 1 Hygiene, Habib Bourguiba University Hospital; 2 Community Health and Epidemiology, Hedi Chaker University Hospital; 3 Social medicine, Faculty of medicine, Sfax, Tunisia

## Abstract

**Introduction:**

Smoking among adolescents remains a major public health issue. Adolescents are especially at risk due to biological, psychological, and social factors. Understanding patterns of cigarette use and early signs of dependence is crucial for developing effective prevention strategies.

**Objectives:**

This study aimed to determine the prevalence of cigarette smoking among adolescents and assess tobacco dependence among conventional cigarette users using the Hooked-on Nicotine Checklist (HONC).

**Methods:**

We carried out a cross-sectional study during the 2024-2025 school year involving adolescents enrolled in middle and high schools in Sfax, Tunisia, using an anonymous self-administered questionnaire. Current cigarette use was defined as having smoked within the past 30 days. A score of ≥1 served as the threshold indicating the beginning of nicotine dependence.

**Results:**

A total of 980 adolescents were surveyed, with 39.2% (n = 384) being male. The prevalence of current conventional cigarette use was 12% (n=118, 95% Confidence Interval (CI95%) = [10.1-14.2]). The median age at initiation was 13 years (Interquartile Range (IQR) = [11-15] years), and the median number of cigarettes smoked per day was 8 (IQR = [3-15] cigarettes per day). According to the HONC test, the median score was 6 (IQR = [2-9]), with 86.2% (n = 100) of adolescent smokers showing signs of nicotine dependence. Nicotine dependence was significantly threefold higher among males (OR = 3.31; p = 0.04). Furthermore, the dependence score was positively associated with the number of years since initiation (Spearman’s rho = 0.45; p < 0.001). Whereas, it was negatively associated with age at initiation (rho = -0.19; p = 0.03).

**Conclusions:**

These results show a high rate of early nicotine dependence among adolescent smokers, especially males and those who started smoking at a younger age. The findings highlight the urgent need for targeted prevention and intervention efforts to reduce tobacco initiation and dependence in this vulnerable group.

**Disclosure of Interest:**

None Declared

